# Probiotics and potential applications for alternative poultry production systems

**DOI:** 10.1016/j.psj.2021.101156

**Published:** 2021-03-26

**Authors:** Rim El Jeni, Dana K. Dittoe, Elena G. Olson, Jeferson Lourenco, Nicolae Corcionivoschi, Steven C. Ricke, Todd R. Callaway

**Affiliations:** ⁎ Department of Animal and Dairy Science, University of Georgia, Athens, GA, USA; † Department of Animal and Dairy Sciences, Meat Science and Animal Biologics Discovery Program, University of Wisconsin, Madison, WI, USA; ‡ Bacteriology Branch, Veterinary Sciences Division, Agri-Food and Biosciences Institute, Belfast, Northern Ireland, United Kingdom; § Faculty of Bioengineering of Animal Resources, Banat University of Animal Sciences and Veterinary Medicine - King Michael I of Romania, Timisoara, Romania

**Keywords:** alternative poultry production, probiotics, gastrointestinal tract, food safety, performance

## Abstract

Concerns over animal welfare continue to be a critical component of law and policies associated with commercial food animal production. Social and market pressures are the driving forces behind the legislation and result in the change of poultry production management systems. As a result, the movement toward cage-free and aviary-based egg production systems has become standard practices. Cage-based systems being replaced by alternative methods that offer a suitable housing environment to meet or exceed poultry welfare needs and require different management, including the ban of antibiotics in poultry diets. For broiler production, pasture- raised and free-range management systems have become more popular. However, challenges remain from exposure to disease-causing organisms and foodborne pathogens in these environments. Consequently, probiotics can be supplemented in poultry diets as commercial feed additives. The present review discusses the impacts of these probiotics on the performance of alternative poultry production systems for improving food safety and poultry health by mitigating pathogenic organisms and improving egg and meat quality and production.

## INTRODUCTION

Commercial poultry production has evolved into a vertically integrated animal industry characterized by its size and production. Currently, to meet the rising demands of the market for meat and eggs, the poultry industry is dependent on the large scale production of meat and egg-type chickens (Dittoe et al., 2020). While conventionally raised broilers are confined to indoor housing for their entire life cycle, public interest in organic and locally grown food sources has led to the pursuit of other management practices (Ricke and Rothrock, 2020). Therefore, a niche for alternative modes of poultry and egg production systems has been created, which has become more widely utilized in recent years. These niches include alternative poultry husbandry practices such as outdoor rearing of pasture flock broilers and cage-free layer aviaries (Mench et al., 2011; Ricke, 2017; Shi et al., 2019; Ricke and Rothrock, 2020). Although the terms free-range and pasture-raised are often used interchangeably, this is not correct. Free-range poultry are poultry reared in a system that allows limited access to the outdoors and is regulated by the United States Department of Agriculture (**USDA**). Whereas pasture-raised, a term not regulated by the USDA, involves birds reared in a system that allows for at least 108 square feet of space outdoors and some sort of shelter (Rothrock et al., 2019).

Antibiotics have been the most widely used additives to improve feed conversion, growth rate, and bird health, increasing both the productivity and profitability in traditional commercial poultry production (Gadde et al., 2017; Lourenco et al., 2019b). However, antimicrobial-resistant bacterial strains originating from animals (Randall et al., 2003; Ungemach et al., 2006; Sweeney et al., 2018) have become an increasingly significant problem over the years, especially regarding transmission via the food supply or direct animal contact (Holmberg et al., 1984; Lanzas et al., 2010; Marshall and Levy, 2011). Other potential food safety threats associated with antibiotic treatment in food animals include an increase in allergic reactions and antibiotic treatment failures in humans (Bruce and Corpet, 1996; Lhermie et al., 2016). As the poultry industry moves increasingly to “No Antibiotics Ever” production systems, and the prophylactic use of antibiotics becomes more restrictive, there is a new ecological niche in poultry flocks that pathogens can occupy (Casewell et al., 2003). Therefore, increasing interest in the application of antibiotic alternatives throughout the commercial poultry industry has occurred. The reduction of antibiotic use has also resulted in the increasing popularity of alternative poultry production systems that do not allow for the use of antibiotics in their production practices (Ricke and Rothrock, 2020). However, feed additives that are acceptable to these alternative production systems are needed to retain health and limit foodborne pathogens (Shi et al., 2019).

While there is an extensive microbial population residing in the gastrointestinal tract (**GIT**) of poultry (Ding et al., 2017; Lourenco et al., 2019a; Feye et al., 2020; Ricke et al., 2020), most of these bacteria are not pathogenic organisms. However, in the presence of certain conditions, specific microorganisms within that population can also behave as opportunistic pathogens. In some foodborne pathogenic bacteria strains such as *Salmonella* Typhimurium or *Escherichia coli*, there has been significant development of antimicrobial resistance in isolates from the poultry intestinal tract (Bager et al., 1999; Fricke et al., 2009; Furtula et al., 2010; Castellanos et al., 2017). These bacteria directly affect human health through the consumption of improperly washed or undercooked eggs or poultry products (Kimura et al., 2004; USDA-FSIS, 2007).

In recent years, attention has been aimed at the indirect contamination routes stemming from the use of poultry litter as a fertilizer. Poultry litter, comprised of feces, feed, wood chips, and other materials, is often used on farms as a fertilizer for the soil and, in turn, resulting in potentially contaminating vegetables, fruits, or water if pathogens are present (Soupir et al., 2006; Locatelli et al., 2017). In addition to contamination of other food commodities and water through the soil, there is concern over the widespread transfer of antibiotic resistance from the prophylactic use of antibiotics in poultry diets. The resistance can occur very quickly, especially if the recipient and donor bacteria are exposed to an environment already containing antibiotic residues (Williams-Nguyen et al., 2016; Castellanos et al., 2017; Poole et al., 2017), and resistance genes bioaccumulate in the human digestive system. Due to the public concern of antibiotics use in poultry feed (KeepAntibioticsWorking.com, 2003; Hao et al., 2014; Hoelzer et al., 2017), banning antibiotics as growth promoters represent a significant challenge for poultry meat production. The dilemma has led to the imperative to identify new approaches or alternatives to remedy this problem (Joerger, 2003; Gaggìa et al., 2010; Marshall and Levy, 2011; Lhermie et al., 2016; Lourenco et al., 2020; Ricke et al., 2020). Subtherapeutic antibiotic use in poultry feed has drawn global concerns for antibiotic resistance in pathogens that are identified as human health risks, resulting in the banning of antibiotics for growth promotion in animal agriculture usage around the world (Casewell et al., 2003; Van Boeckel et al., 2015; Ricke et al., 2020).

Alternatives to antibiotics have been progressively introduced into animal agriculture (Seal et al., 2013; Gadde et al., 2017; Ricke et al., 2020). Antibiotics have been replaced by products that proved to be viable in alternative poultry production schemes and include organic production, such as enzymes, organic and inorganic acids, probiotics, prebiotics, synbiotics, herbs, and essential oils (Callaway et al., 2013; Grilli et al., 2015; Callaway et al., 2017; Shi et al., 2019). In the present review, the focus will be on the use of “probiotics” as a broad approach to reducing pathogens (including antimicrobial-resistant organisms) in alternative poultry production systems such as free-range and pasture-raised systems.

In some ways, similar to conventional poultry production, several factors can impact alternative production systems, and the inclusion of the appropriate probiotic may improve efficiency, reduce morbidity, reduce mortality, reduce environmental pollution, and enhance food safety. However, besides the shared impacts associated with the two systems, alternative production operations such as partial outdoor, cage-free, and pasture flocks have additional influential factors unique to the environments these birds are experiencing. When used in alternative poultry systems, probiotics would be expected to have similar favorable effects to antibiotics used in conventional poultry production systems. After egg hatching, their administration in feed has shown a beneficial impact on improving animal health. This review will illustrate the critical role of probiotics to limit foodborne pathogen colonization, prevent some common diseases that can harm free-range poultry flocks and improve product characteristics of alternative poultry meat and eggs.

## PROBIOTICS – GENERAL CONCEPTS AND DEFINITIONS

The modern concept of “probiotics” was first developed by Ilya Metchnikoff, who noted that human populations in rural parts of Bulgaria experienced a higher life expectancy than average, which was correlated with drinking large quantities of fermented milk products (Metchnikoff, 1907; Vasiljevic and Shah, 2008). Metchnikoff suggested a select type of microorganism existed in the milk product that altered bacterial fermentation in their intestines (Metchnikoff, 1907). The particular bacteria or probiotic organism Metschnikoff based his theory on was identified as *Lactobacillus bulgaricus* from “podkvassa,” which was the culture starting agent in the production of Bulgarian “kiselo mleko” (Grigoroff, 1905; Vasiljevic and Shah, 2008).

The term “probiotic” resulted from an observed phenomenon between cocultured organisms where one of the microorganisms produced growth-promoting substances that stimulated the growth of other microorganisms (Lilly and Stillwell, 1965; Tannock, 1997). The definition of probiotic refers to a ‘live microbial feed supplement which beneficially affects the host by improving its intestinal microbial balance’ (Fuller, 1989); this being a direct refinement of previous work through an emphasis on the “live cell” component of probiotics (Lilly and Stillwell, 1965). In recent years, some of these live probiotic cultures have begun to be described by the general term “Eubiotics,” which is related to the Greek word “Eubiosis,” referring to an optimal microbiota balance in the GIT (Miniello et al., 2017; Yaşar et al., 2017). Although probiotics have been employed in a broad spectrum of usage to improve and maintain human health (Suvarna and Boby, 2005; De Preter et al., 2011; Wang et al., 2016), food animal agriculture has primarily utilized probiotic supplementation (known as “Direct-Fed Microbials”). In agriculture, probiotics are used to increase feed efficiency, growth promotion (Jin et al., 1997), and foodborne pathogen reduction (Nahashon et al., 1994b; Krehbiel et al., 2003; McAllister et al., 2011).

Historically, probiotic utilization in the poultry industry has been viewed as an alternative to antibiotics that promoted a “healthy” intestinal microbiota (Mátéová et al., 2008; Katoch et al., 2013; Vandana et al., 2013), improved growth rates, limited animal diseases (Gorbach, 2000; Nava et al., 2005; Ghareeb et al., 2008; Callaway et al., 2012), and inhibited foodborne pathogen growth (Patterson and Burkholder, 2003; Lutful Kabir, 2009; Murate et al., 2015; Olnood et al., 2015; Penha Filho et al., 2015; Upadhaya et al., 2016). There are currently a vast number of probiotic products in the marketplace being promoted to improve poultry production efficiency, health, and safety. The hypothetical ideal characteristics of a probiotic have been described in detail by Patterson and Burkholder (2003) and will not be discussed further in the current review.

A broad range of fungi, protozoa, and bacteria have been tested for probiotic capabilities and have been utilized in field trials; however, only a select few have reached industry level availability (Vasiljevic and Shah, 2008). Much of the limitation in selecting probiotic cultures involves the requirement of being on the “Generally Recognized as Safe” (**GRAS**) list to reduce the regulatory hurdles encountered during commercialization. Modes of action of probiotics vary from culture to culture yet fall into a relatively small number of categories (Khan and Naz, 2013; Buntyn et al. et al., 2016). Among the mechanisms suggested include production of short chain fatty acids (**SCFA**), bacteriocins and other inhibitory substances by probiotic organisms (Joerger, 2003; Patterson and Burkholder, 2003). Probiotic competition for adhesion sites on the GIT epithelium that prevents physical binding by the pathogen can be a factor as well (Nurmi et al., 1992; Wray and Davies, 2000, Schneitz, 2005). In addition, stimulation of the host immune system may also play a role (Kogut and Klasing, 2009; Kogut and Swaggerty, 2011). However, given the complexity of the microbial consortia in the poultry GIT, the mode of action of probiotics is the most likely a combinatorial effect of all the previously mentioned modes of actions as well as other less defined mechansism, rather than a single action. Thus, most of the benefits of probiotic feeding (e.g., improved growth efficiency and reduced foodborne pathogens) may be the result of a complex array of mechanisms.

## IMPACT OF PROBIOTICS ON POULTRY PRODUCTION

Probiotics are often fed to poultry to potentially increase feed intake and nutrient retention (Ghareeb et al., 2012). Many have proven to be consistently beneficial by eliciting positive impacts on GIT morphology, microbial populations, nutrient absorption, intestinal barrier function, antioxidative capacity, apoptosis, and immune responses, ultimately promoting GIT health and broiler production performance (Callaway et al., 2011; Rodjan et al., 2018; He et al., 2019; Wu et al., 2019). The efficacy of probiotics in poultry, and food animals, in general, is often based on feed intake (**FI**), body weight gain (**BWG**), feed conversion rate (**FCR**), and the health or welfare status of the animals by reducing the frequency of morbidity and mortality during certain critical phases of production, such as dietary stress (change of diet, rations rich in concentrates) and health stress (e.g., animal density and other factors) (Yeo and Kim, 1997; Callaway et al., 2011; Katoch et al., 2013; Vandana et al., 2013; Palamidi et al., 2016; Yazhini et al., 2018; Vase-Khavari et al., 2019).

Treatment with probiotics has resulted in an increase in serum protein, albumen, and a reduction of total serum cholesterol and triglycerides in broilers (Yazhini et al., 2018). Following probiotic administration to broilers, the reduction of cholesterol and fat content in the breast and thigh meat was observed (Hossain et al., 2012). Additional studies report increased fatty acids in broiler meat and higher levels of vitamin E and other nutrients (Trembecká et al., 2016). Access to pasture or insects may also contribute to the organoleptic quality of the product, which could impact market acceptability for pasture reared and free-range poultry products (Ponte et al., 2008; Hammershøj and Johansen, 2016; Ruhnke et al., 2018). For example, Al-Qazzaz et al. (2016) reported improved FCR, egg production, egg weight, shell thickness, shell weight, egg yolk color, fertility, and egg mass when commercial layers were fed black soldier fly larvae in commercial diets (Al-Qazzaz et al., 2016).

Supplementation of free-range and pasture-raised flock diets with probiotics has the potential to not only improve production and organoleptic quality of poultry and poultry products, but reduce the environmental burden of rearing poultry. As such, retaining dietary nutrients through probiotic supplementation represents a potential opportunity to improve growth performance and reduce the environmental (pollutant) burden from poultry production. One major concern of agriculture production, such as poultry production, is its contribution to the build of phosphorus, potassium, and nitrogen in the soil and contribution to eutrophication of waterways (Dittoe et al., 2018). The primary reason poultry production contributes to phosphorus leaching is the loss of available phosphorus by phytate. Phytate is a phytochemical that binds phosphorous, making it unavailable to the animal; thus, there is significant interest in using the microbial enzyme phytase to retain available phosphorus (Perney et al., 1993; Nahashon et al., 1994a; Kim et al., 2007). The supplementation of probiotics in poultry diets has demonstrated a phytate degradation mechanism. Through the inclusion of recombinant *Lactobacillus* cultures in poultry diets, broilers experienced improved weight gain resulting in reduced production cost and environmental impacts (Askelson et al., 2014). In some cases, the use of probiotic additives has lowered the quantity of nitrogen in waste effluent, which potentially represents a gain in feed efficiency and reduced nitrogen requirements in diet formulations, resulting in a reduction of leached nitrogen on the farm and the surrounding environment (Rotz, 2004; Applegate et al., 2010).

Not only do probiotics have the potential to reduce nutrient requirements by enhancing nitrogen and phosphorus utilization, but some probiotics have also demonstrated significant immunomodulatory potential (Apata, 2008; Brisbin et al., 2011; Kogut and Swaggerty, 2011; Cox and Dalloul, 2015; Palamidi et al., 2016). Protection against pathogens and facilitating digestion and nutrient utilization may be resolved by modulating the immune response (Rinttilä and Apajalahti, 2013). These benefits can be accomplished by enhancing the innate and acquired immunity of poultry (Swaggerty et al., 2019). Specifically, Swaggerty and colleagues (2019) suggest that influencing the innate immunity through modulating the proliferation of macrophages, heterophils, and B1-type lymphocytes is more advantageous than stimulating the acquired immunity. However, more research is needed to delineate such differences.

Previously, the weekly oral supplementation of lactic acid bacteria (**LAB**), such as *Lactobacillus acidophilus, Lactobacillus reuteri*, and *Lactobacillus salivarius,* in commercial broiler chicks resulted in improved antibody- and cell-mediated immunity (Brisbin et al., 2011). Brisbin et al. (2011) demonstrated that after immunizing birds with sheep red blood cells (**SRBC**), keyhole limpet hemocyanin (**KLH**), Newcastle disease virus vaccine, and infectious bursal disease virus vaccine at 14 and 21 d of age, the supplementation of *L. reuteri* was able to modulate the chicken immune system, whereas, *L. acidophilus* and *L. salivarius* were not. Overall, Brisbin et al. (2011) concluded that the oral treatment of broilers with LAB could stimulate the immune system, but they may vary in their ability to modulate the immune response.

Probiotics have demonstrated beneficial effects on the immune system to control inflammation in poultry by interacting with the intestinal epithelial and immune cells. In order to select strains for potential use as probiotics, the immunomodulatory properties of LAB were tested in vitro to determine their ability to survive acidic conditions (pH 2.5) and bile salts (0.1 to 1.0%), reduce 6 pathogens, and adhere to Caco-2 cells (Feng et al., 2016). Subsequently, Feng and colleagues selected six LAB strains from the in vitro screening. Three of those strains (*Lactobacillus plantarum* PZ01, *L. salivarius* JM32, and *Pediococcus acidilactici* JH231) reduced the levels of lipopolysaccharide-induced TNF-α factor (**LITAF**), IL-1β, IL-6, and IL-12 and increased IL-10 in the serum *in vivo* during a *Salmonella* challenge in commercial broilers. In addition, the influence of 4 probiotics (*Lactobacillus, Bifidobacterium, Enterococcus*, and *Pediococcus* strains) in broilers was evaluated by Mountzouris et al. (2007). Conventionally-reared broilers received a corn-soybean basal diet with or without a probiotic administered in the feed or water supply. Those broilers fed diets supplemented with the probiotic cocktail in the feed or water exhibited significantly higher specific activities of two glycolytic enzymes associated with gut modulation and immune stimulation, α-galactosidase and β-galactosidase, compared with those fed the control and antibiotic supplemented diets.

In summary, much of these benefits involve a hypothesized modification of the intestinal ecosystem, which often has yielded widely variable results among studies. Much of the impact is dependent upon several parameters including the strains of microorganisms used, probiotic concentrations in the feed, the interaction of probiotics with individual components of the ration, interactions with the indigenous microbiota, bird age, and the nutritional and health status of the birds (Cui et al., 2017).

## ZOOTECHNICAL PROBIOTICS, FOOD SAFETY, AND POULTRY HEALTH

Probiotics exert antibacterial activities through the direct competitive opposition and indirect exclusion (Nurmi et al., 1992; Kizerwetter-Swida and Binek, 2009; Callaway et al., 2017) against various poultry foodborne pathogens such as *Salmonella* and *Campylobacter* spp. (Line et al., 1998; Siemon et al., 2007; Willis and Reid, 2008; Higgins et al., 2010; Knap et al., 2011; Mortada et al., 2020). Commercial probiotics such as Lavipan (*Lactococcus lactis, Carnobacterium divergens, Lactobacillus casei* and *plantarum,* and *Saccharomyces cerevisiae*) have been shown to reduce *Campylobacter* spp. invasion of the GIT under commercial field conditions resulting in the mitigation of *Campylobacter* population in the GIT and the resulting carcasses after processing (Smialek et al., 2018). The supplementation of Lavipan in broiler diets demonstrated an increase in body weight gain and a decrease in coliform proliferation of broilers (Smialek et al., 2018). In addition, the rate of lactobacilli growth in the crop increased at the beginning of the first day of administration (Smialek et al., 2018). However, no significant decrease in the *Campylobacter* populations was observed in the cecum (Smialek et al., 2018), suggesting this probiotic activity occurs primarily in the small intestine (Pascual et al., 1999) or was unable to reach the hindgut. Broilers orally gavaged with *L. salivarius* exhibited effective prevention of *Campylobacter jejuni* colonization in broiler intestines (Saint-Cyr et al., 2017). Probiotics are also active against less frequent foodborne pathogens that affect poultry health and poultry products, such as *E. coli* (Chang and Chen, 2000; La Ragione et al., 2001; La Ragione et al., 2004), *Staphylococcus aureus* (El‐Kholy et al., 2014), *Yersinia enterocolitica* (Bujalance et al., 2014), *Clostridium perfringens* (Zhou et al., 2016; Li et al., 2018; Ramlucken et al., 2020), and *Listeria monocytogenes* (Olnood et al., 2015).

Necrotic enteritis (**NE**) caused by *C. perfringens* is a poultry production disease that can lead to morbidity and mortality in chickens (Sokale et al., 2019). The onset of NE has been successfully reduced in poultry by numerous probiotics (La Ragione et al., 2004; Taheri et al., 2010; Olnood et al., 2015; Bortoluzzi et al., 2019; Sokale et al., 2019). Several bacterial strains (*Bacillus subtilis* and *licheniformis, Enterococcus faecium, Lactobacillus acidophilus, Butyricicoccus pullicaecorum*) have been used as probiotics against subclinical necrotic enteritis (SNE) (Eeckhaut et al., 2016; Li et al., 2018; Sokale et al., 2019; Wu et al., 2019). If populations of *C. perfringens* can be kept low in the GIT by competitive exclusion, poultry well-being and production can be improved.

Aflatoxin, a family of toxins produced from certain molds (*Aspergillus flavus* and *A, parasiticus*), poses a significant risk to poultry production because of the direct toxicity which impacts bird mortality and morbidity throughout the production cycle (Otim et al., 2005; Hussain et al., 2010; Tarus et al., 2019). Because of the sporadic nature of aflatoxin production and accumulation in the diet of poultry, it is difficult to predict and detect in many cases (Maciorowski et al., 2007). However, several probiotics have demonstrated direct antiaflatoxin impact in several food animal species (Baines et al., 2013; Intanoo et al., 2018), including broilers and hens (Ma et al., 2012; Motawe et al., 2014).

## PROBIOTICS FOR PASTURE FLOCK BROILERS

Minimal research has been published on the effect of probiotics on pasture flock broilers. In general, it has been proven that probiotics have a positive impact on different production parameters when administered as feed additives to broilers over the entire rearing period (Rowghani et al., 2007; Nunes et al., 2012). Pelícia et al. (2004) evaluated the effects of a no treatment control (1) and three commercial treatments: the combined supplementation of the probiotic “Colostrum avus” (*Enterococcus sp.*) of bacterial origin and the prebiotic “Simbiotico plus”(a mannan oligosaccharide from the cell wall of *Saccharomyces cerevisiae*) (2); the combination of a probiotic and prebiotic of yeast origin, Levucell SB 20 (cell wall components and live cells of *Saccharomyces cerevisiae*) (3); and a mixture of 2 and 3 (4). The treatments were tested for efficacy based on the performance, development of the digestive system, carcass yield, and meat quality of a free-range strain of broiler chickens (ISA S757-N Label Rouge). A total of 560 birds were reared in commercial production until 35 d of age, where they were provided limited access to the outdoors. Pelicia et al. (2004) observed that birds supplemented with dietary treatment 2 had reduced mortality up to 35 d of age compared to the control birds, likely due to probiotic bacteria GIT colonization, which conferred higher resistance in the birds. Significant weight gain from 64 to 77 and 64 to 84 d of age was observed in birds fed treatment 2 compared to the control birds. Moreover, there were significant differences among treatments for carcass yield. Birds supplemented with treatment 2 and 3 exhibited higher total carcass yield than control birds, but there was no effect of treatment on the yields of the breast, breast meat, leg, leg meat, wings, and back. Pelícia et al. (2004) also did not detect performance differences among treatments; however, in the later phases, the addition of treatment 2, probiotic and prebiotic from bacterial origin, to poultry diets increased weight gain compared to the control group. The addition of probiotics and prebiotics of different origins did not affect the digestive tract and affiliated organs (liver, proventriculus, gizzard, pancreas, duodenum, jejunum, ileum, and cecum).

The efficacy of utilizing prebiotics and probiotics (85% of mannooligosaccharides and 10^6^ CFU of *Enterococcus* spp. per gram of product) was evaluated on *Salmonella* incidence in the carcasses of a free-range strain of broilers (Naked Neck Label Rouge; Takahashi et al., 2005). During trial two of the study, at 35 d of age a total of 128 male chicks were given access to outdoor paddocks (3 m^2^/bird). The GIT morphology was not affected by the addition of probiotics and prebiotics; however, the incorporation of probiotics and prebiotics in infected flocks with *Salmonella* Enteritidis significantly reduced subsequent carcass contamination with *Salmonella* spp. During trial one of the study, birds were supplied diets containing probiotics and prebiotics and either confined indoors or reared in a free-range system. Those supplemented with the diets containing prebiotics and probiotics and the confined birds showed better performance, carcass yield, and meat quality than the birds of the other treatments. Birds fed diets supplemented with probiotics and prebiotics also showed higher body weight and weight gain, whereas feed intake was increased in the control birds. Birds fed the diet containing probiotics and prebiotics had greater height and length of breast fillets. Pelícia et al. (2004) also reported that meat quality characteristics were similar between a free-range strain of broiler chickens fed diets with different additives (probiotics, prebiotics, and antibiotics) and given limited access to the outdoors at 35 d of age.

Free range chickens may also be a viable source of probiotics for commercial poultry production. For example, Neveling et al. (2020) evaluated probiotic strains (*Lactobacillus gallinarum, L. johnsonii, L. salivarius, L. crispatus, Enterococcus faecalis*, and *Bacillus amyloliquefaciens*), isolated from the GIT of 25 healthy free-range broilers (crop, proventriculus, ventriculus, duodenum, small intestine, cecum, and large intestine) from Hermanus, Graafwater, Fisantekraal and Grabouw in the Western Cape of South Africa. They demonstrated the in vitro tolerance of the probiotic candidates toward simulated GIT conditions such as acidic conditions and bile salts. They produced hetero-exopolysaccharides, which aided their vitality in the GIT harsh conditions, and could be utilized for the formation of biofilms. *Bacillus amyloliquefaciens* produced extracellular amylase and antimicrobial lipopeptides. Amylase, an enzyme that increases starch degradation, has the potential to improve poultry growth performance. *L. salivarus, L. crispatus*, and *L. johnsonii* produced hydrogen peroxide, which has the ability to inhibit the colonization of oxidative-sensitive bacteria. *B. amyloliquefaciens* and *E. faecalis* were capable of producing phytase, an enzyme that can increase nutrient availability in feed, subsequently improving the growth performance of poultry. In addition, *L. crispatus* and *E. faecalis* used in the study produced the bile salt hydrolase, which indirectly leads to a lowering of cholesterol effects. Each strain possessed specific probiotic properties, and the combination of these strains, when provided to commercially reared broilers, exhibited the best protection against pathogens (Neveling et al., 2020).

The supplementation of probiotics isolated from the intestines of free-range chickens also has the potential to improve the meat composition and health of the birds by modulating the GIT microbiota (Aziz et al., 2020). For example, MiaClost, a commercial poultry water supplement consists of two probiotic bacteria, *E. faecium* and *B. subtilis* spores that were originally isolated from the GIT of free-range chickens and supplemented at a rate of 100 g per 1,000 L (MIAVIT GmbH, Essen (Oldenburg), Germany). In fact, the supplementation of the probiotic MiaClost to the drinking water of commercial broiler chicks (no access to outdoors) led to a decrease in the percentage of moisture and an increase in the proportion of protein in the breast and thigh meat at 42 d of age (Aziz et al., 2020). Additionally, there was a significant effect on the fat and ash of the two cuts of meat when birds were supplemented with MiaClost. However, there was no influence on the pH of breast and thigh meat. The probiotic supplementation of 0.160 g of MiaClost per liter of drinking water did contribute to a significant increase in water holding capacity in the breast. In contrast, the supplementation of 0.175 g of MiaClost per liter of drinking water resulted in a decrease in the water holding capacity in the breast (Aziz et al., 2020). In addition to improving meat quality parameters, the supplementation of MiaClost in the drinking water demonstrated the potential to improve growth parameters as well (Aziz et al., 2020). Aziz et al. (2020) reported that the supplementation of 0.160, 0.175, and 0.190 g/L to commercially reared broilers (no access to the outdoors) improved the feed intake, body weight gain, feed conversation ratio, and the intestinal morphology. During the performance study, there was no difference between the supplementation of 0.175 and 0.190 g/L, whereas the supplementation of 0.160 g/L was optimal to increase the meat quality but the higher doses resulted in decreased meat quality (Aziz et al., 2020). Therefore, the optimal concentration may be 0.160 g/L of MiaClost although the producer of the product suggests a dosage of 0.1 g/L. Overall based on the outcomes of these studies, it appears that the responses to probiotic supplementation in free-range broilers are not inconsistent. In general, probiotics may be more effective in broilers experiencing stress, possibly due to the presence of unfavorable organisms, extremes in ambient temperature, diseases, crowding, and poor management, which can occur in both conventional and alternative production systems. In addition, free-range chickens may harbor probiotic candidates that have utility in conventional poultry production.

## PROBIOTICS FOR PASTURE, CAGE-FREE, AND ORGANIC LAYING HENS

Another potential niche for probiotics is within the pasture, cage-free, and organic laying hens. Over the past few years, organic poultry production has increased by 23% from 2015 to 2016 with U.S. farms and ranches selling over $7.6 billion in certified organic commodities in 2016 (US Egg and Poultry, 2020). In addition, eggs were the second highest certified organic commodity bringing in over 816 million dollars in 2016 an 11% increase from 2015 (US Egg and Poultry, 2020). Currently, 5.8% (19.4 million) of layer hens are used for organic egg production, 17.8% (60 million) of layer hens are used for cage-free egg production, and 76.4 % (257.1 million) of layer hens are used for conventional egg production (United Egg Producers, 2020). On average, 287.1 eggs per capita were consumed in the US in the year 2019, making eggs the most consumed animal protein within the US (US Egg and Poultry, 2020).

Eggs are likely so popular among consumers of the U.S. and other countries as they are one of the most cost-effective sources of animal proteins and lipids and can be utilized in a variety of food products (United Egg Producers, 2020). Of the 275 million eggs produced in 2019, 60.1 % (165.5 million cases) eggs were sold as retail shell eggs, 30.1% (82.9 million cases) were used for further processed foods, 7% (19.3 million cases) were used in food service, and the remaining 2.8% (7.6 million cases) were exported out of the U.S. (United Egg Producers, 2020). One major interest of egg production is the nutritional aspect of eggs, such as antioxidant components (Lu and Baker, 1986). Many of the antioxidant components of eggs are within the egg yolk, mainly phosvitin which chelate Fe (III) ions (Lu and Baker., 1986). Phosvitin also provides protection against the formation of iron-catalyzed hydroxyl radicals (Lu and Baker., 1986). These antioxidants could be used to prevent colorectal cancer since iron-modulated oxidative stress is implicated in this pathology. Adding to consumers’ market preferences, parameters in the shell, such as color and strength, are also of value (Żakowska-Biemans and Tekień, 2017).

With the interest in pasture-raised poultry, there is a potential for egg characteristics to improve as poultry that have access to high-quality pastures could potentially produce eggs that contain more beneficial qualities than those without access to pastures. According to Karsten et al. (2010), eggs of pasture-raised hens are distinguishable from the eggs of sister hens fed only a commercial mash diet in conventional cages for 6 wk. Eggs of the hens consuming grasses in the pasture system had 23% more vitamin E than eggs of hens grazed clover. Overall, eggs produced from pastured hens contained twice as much vitamin E and long-chain omega-3 fats, a 2.5-fold increase in total omega-3 fatty acids, and less than half the ratio of omega-6:omega-3 fatty acids in comparison with the eggs of the caged hens (Karsten et al., 2010). Although the amount of vitamin A per egg did not differ, vitamin A accumulation was 38% higher in the pastured hens’ eggs than in the caged hens’ eggs (Kartsen et al., 2010). However, pastured hens may have lower body weight and egg production than caged hens unless supplemented sufficiently to meet their dietary energy and crude protein needs (Kasten et al., 2010).

There is also a precedent with other avians such as quail to support the benefits of probiotics on egg production. For example, Manafi et al. (2016) assessed the performance and GIT health of Japanese quails (*Coturnix Coturnix japonica*) when provided diets supplemented with *B. subtilis*. It was demonstrated that supplementing the Japanese quail diets with probiotics such as *B. subtilis* improved egg production and egg weight (Manafi et al., 2016). In addition, GIT crypt depth was reduced, villi height and villi to crypt ratio were increased, and *Salmonella, E. coli*, and total coliforms were reduced (Manafi et al., 2016).

Other probiotics, such as LAB, have also been shown to improve layer performance. Some of this improvement could be related to improved GIT health. For example, studies have shown that probiotics may prevent reproductive tract lesions within poultry, potentially increasing layer performance (Shini et al., 2013). These improvements could also be evident from more efficient nutritional responses of layers. Lokapirnasari et al. (2019) determined that the use of probiotics, *Bifidobacterium* spp. and *L. casei,* could improve the growth performance and egg production in organic laying hens. Probiotic administration (0.5% *Bifidobacterium* spp. + 0.25% *L. casei*) was implemented at intervals of 1, 2, 3, and 4 wk in 180 laying hens (Lohmann) at 30 wk of age. Probiotic supplementation in the diets during wk 1 and 2 exhibited the lowest feed intake, with the highest egg weight in the 1st wk of administration. The 0.5% *Bifidobacterium* spp. + 0.25% *L. casei* in the 2nd, 3rd, and 4th wk revealed no detectable differences compared to the use of 0.1% antibiotic growth promoters (AGP) in the 2nd and 3rd wk (Lokapirnasari et al., 2019). The results showed that supplementing 0.5% *Bifidobacterium* spp. + 0.25% *L. casei* and 0.1% AGP yielded relatively similar results. The lowest FCR was observed for the treatment of 0.5% *Bifidobacterium* spp. + 0.25% *L. casei* used for 1 to 4 wk (Lokapirnasari et al., 2019). The provision of 0.5% *Bifidobacterium* spp. + 0.25% *L. casei* for 1 to 4 wk yielded FCR results significantly different from the administration of 0.1% AGP for wk 1, 2, 3, and 4 (Lokapirnasari et al., 2019). High FCR results were found in controls when AGP and probiotics were not provided. The low FCR was caused by administering probiotics that can reduce feed consumption, while egg production remained high. *Lactobacillus* is one of the dominant groups of microorganisms located in the duodenum in chickens and has been associated with better feed efficiency (Bjerrum et al., 2006; Wang et al., 2014). This improvement in feed efficiency and reduction in FCR by additional probiotics could be related to the enhancement of the metabolism of digestion processes and the utilization of nutrients due to its supplementation (Lokapirnasari et al., 2019).

Incorporating probiotics as feed additives may also increase nutrient absorption due to an improved intestinal mucosal structure. Lokapirnasari et al. (2019) determined that the supplementation of LAB probiotics in diets has the potential to improve the mucosal structure in the GIT, thereby suppressing pathogenic bacteria growth, resulting in improved egg production. An optimally functioning GIT can help improve the metabolic process and absorption of nutrients needed by the host (Lokapirnasari et al., 2019); however, this may depend on the type of probiotic. For example, two different probiotics (*L. acidophilus* or *B. subtilis*) were compared in organic laying hens fed corn-soybean cake-based diets (Forte et al., 2016). Subgroups of hens at 18 wk, 5 mo, and 7 mo were euthanized, and small intestinal duodenum sections were subsequently removed for morphological and morphometric examination. Ileal and cecal contents were enumerated for *E. coli*, coliforms, *Enterococci, Staphylococci, Clostridium* spp., total anaerobes, *Bifidobacterium*, and lactobacilli. Duodenal morphological and morphometric responses did not appear to be influenced by either probiotic, but microbiological differences did occur (Forte et al., 2016). In general, *L. acidophilus* probiotic-fed hens yielded the lowest populations of *E. coli*, coliforms, *Staphylococci*, while both probiotics reduced *Clostridium* spp. Both probiotics increased lactobacilli and *Bifidobacterium*. Interestingly, *Bifidobacterium* were less predominant in the ileum of older birds fed *L. acidophilus* than birds receiving *Bacillus.* However, *Bifidobacterium* were higher in the cecum of older birds fed *L. acidophilus* than birds fed the *Bacillus* probiotic (Forte et al., 2016). These results suggest that some competition may occur between *Bifidobacterium* and lactobacilli in the ileum, potentially shifting certain LAB groups into the cecum. However, total anaerobes were minimally impacted by either probiotic (Forte et al., 2016). Further studies involving complete microbiome characterization of both sections of the layer hen GIT along with lactic and SCFA quantitation may reveal whether this is occurring. It is also possible that specific *Lactobacillus* spp. could vary depending upon the region and locations in the GIT (Adhikari and Kwon, 2017). Therefore, in addition to microbiome characterization, specific detection assays for each probiotic strain are warranted to better understand the establishment of each respective probiotic isolate and its respective colonization site.

## INFLUENCE OF PASTURE FORAGES

The ability of probiotics to influence birds’ responses in alternative poultry production systems can be affected by other factors related to how these birds are raised, such as the availability of pastures for potential grazing (Sossidou et al., 2015). Hypocholesterolemic and anticarcinogenic compounds present in forage can lead to improved meat quality (Angelovičová et al., 2013), but also have the potential to negatively impact consumer sensory perceptions. Forages such as alfalfa naturally generate molecules, including triterpenoid and steroidal glycosides, referred to collectively as saponins (Rao and Gurfinkel, 2000; Francis et al., 2002). These are bioactive compounds possessing hypocholesterolemic, anticarcinogenic, and anti-inflammatory properties, along with antioxidant activities (Rao and Gurfinkel, 2000; Francis et al., 2002). Researchers demonstrated that the addition of polysavone, a natural extract from alfalfa, to broiler diets has the potential to increase the serum anti-Newcastle disease virus hemagglutination inhibition antibody titer and lymphocyte proliferation (Dong et al., 2007). In addition, polysavone supplementation decreased abdominal fat deposition and enhanced immune parameters with no negative impact on Arbor Acre broilers (Beijing Huadu Broiler Co., Beijing, China) (Dong et al., 2007). However, polysavone has been shown to reduce growth performance, reduce abdominal fat, and increase breast and drumstick meat yield in organic broilers when housed in an indoor pen with access to a grass paddock compared to the control birds (Castellini et al., 2002).

Within the responses of birds to these forage sources and potential metabolites, it would be interesting to know if probiotics could play a role in enhancing beneficial effects of individual plant metabolites and/or countering the more negative aspects of some metabolites via fermentation activities that detoxify certain chemical moieties. Thus, probiotic benefits could be translated to further improvements in total cholesterol, triglyceride levels, and immunological parameters (Jin et al., 1998). Dietary adaptation in combination woth probiotics may also offer the potential to improve poultry meat flavor in pasture-raised birds. However, little is known about how antimicrobials and probiotics affect the taste, odor/flavor, appearance, and texture of poultry products (Lutful Kabir, 2009). More research on the influence of pasture foraging and probiotics on the sensory qualities of free-range poultry products is needed to design optimal nutritional management strategies for maximizing any benefits attributable to these factors. Since production parameters are somewhat unique with pasture-flock birds as well as potential breed differences other sensory and meat properties may respond in other ways not typically associated with conventional broilers.

## FUTURE DIRECTIONS

As alternative poultry production practices and their corresponding markets develop accompanied by expansion in consumer demand for more of these alternative poultry products, an increase in the need for pathogen prevention and efficient production management options will become more critical. Besides the typical production concerns, alternative poultry production practices must also consider environmental impacts and animal welfare concerns unique to their operations. Without antimicrobials or chemical treatments, more natural protective methods such as probiotic supplementation are needed to improve growth efficiency, product quality, and food safety. The use of probiotics has been shown to impact the microbial population of the GIT, improve degradation of feedstuffs, elicit immunostimulatory properties, and exclude animal health agents and foodborne pathogens in conventionally produced flocks. Presumably, much of this also holds for alternative poultry production. Although individual probiotics may vary, they have been shown to benefit alternative poultry production systems through various mechanisms. However, poultry production responses are still somewhat unpredictable and identification of specific probiotic mechanisms in the GIT continues to be elusive. This remains true for conventional poultry production and likely holds true for alternative poultry production systems as well. In all cases a better understanding of the functionally of probiotics once they enter the GIT is needed.

To specifically improve the utility of probiotics in alternative poultry production, further understanding on how probiotics function and influence the GIT microbial populations in birds raised under alternative poultry production conditions is needed. As such, there is a need for additional research to be conducted under alternative poultry production conditions to determine specific immune system stimulation mechanisms mediated by probiotic administration and if these alternative production systems have unique impacts on bird responses attributable to these environmental conditions. Further investigations should focus on studying the bidirectional interactions between the microbial population and the host, particularly the probiotic impact on host tissue-specific effects and the intestinal digestive process, which could improve the use of these compounds in poultry. Advances in metagenomics, nutrigenomics, and metabolomics should shed new light that will lead to a more comprehensive understanding of these interactions. Some of the critical issues to be considered when developing food animal probiotics include strain characterization, end products from multiple dietary sources (e.g., cereal grain versus pasture), quality control, dose optimization, heat stability, aerotolerance, and potential lateral gene transfer from probiotics to native microbiota (especially regarding antimicrobial resistance or toxin genes). Therefore, developing a database of these responses in vitro and *in vivo* of birds being raised under alternative poultry production conditions will provide the means to optimize probiotic applications specifically for these birds.

Developing this database for alternative poultry production systems has other advantages as well. A key area of potential future research is the screening of pasture flock birds for probiotic isolates that could be highly effective in conventional poultry production systems. Some of these probiotic candidates may possess specific or unique properties related to their free-range source that promote a certain level of robustness to counter environmental and other stresses encountered in commercial production systems. Likewise, differences in diets and access to high fiber sources may potentially select for GIT microorganisms that when administered as probiotics could contribute to the breakdown of less digestible components of cereal grain diets. Given the wide range of alternative poultry production system environments, along with dietary and breed differences, the resulting diversity may offer a very wide range of candidate probiotic isolates that could provide multiple benefits beyond the typical probiotic cultures currently in use for conventional commercial poultry production.

## CONCLUSIONS

Alternative poultry production systems continue to expand in response to increasing market demand. As alternative poultry production capacity increases, the need for feed additives that can protect bird health, improve performance, and limit foodborne pathogen establishment becomes more important as a management tool. Several feed amendments are available for potential use in alternative poultry production operations including phytochemicals, prebiotics, and probiotics among others. The use of probiotics (e.g., eubiotics) may be one of the more optimal solution that offers several benefits and a wide range of applications in different production conditions and diets (Gaggìa et al., 2010). Certainly there is precedent for their commercial application based on their use in conventional poultry production and it would appear that they have utility in free-range poultry and other alternative production systems as well. The probiotic studies conducted thus far would support the premise that probiotics may provide some benefits to alternative poultry management outcomes.

However, more research is needed to better understand how probiotics mediate their benefits to the bird host. It is believed that probiotics can impact the GIT microbial population to some extent such as limiting foodborne pathogen colonization. However, the impact on the overall GIT microbial populations is much less clear. In recent years, numerous advanced “omics” studies have been carried out to expand an understanding of probiotic impacts but these results remain highly variable. Probiotic impacts (including extent, magnitude, and duration) on microbial community responses depend on many factors that are not entirely understood at this time. The factors include species of bacteria and corresponding quantities administered, guild function of bacteria utilized, GIT ecological niche occupancy, microbial population redundancy within niches, metabolic products in the commercial probiotic (e.g., amino acids, vitamins), ration, type of target animals (e.g., production system, strain, age) and rearing conditions (e.g., presence or absence of environmental and production stresses).

Some probiotic impacts in the GIT are becoming more defined. For example, stress results in GIT dysbiosis, which allows pathogens (both animal and foodborne) to enter the animal and obtain a foothold in the GIT or systematically within the animal. Studies have found that some probiotics indeed improve outcomes in poultry undergoing stresses due to alleviating the selective pressure of dysbiosis being introduced into the GIT. However, these subtle and limited impacts may not encompass all the criteria desired for optimal health or be detectable in standard production metrics such as body weight gain, FCR, or meat quality. Therefore, care must be taken in which probiotic is selected to determine overall goals for the usage of the probiotic and which parameters will be used to determine success. This may be particularly true for alternative poultry productjon systems where the potential influential factors may not only be more complex than conventional operations but more variable due to the fluctuations in environmental exposure that these birds encounter. Optimizing the selection of probiotics may help the bird in alternative poultry production systems to achieve some stability to effectively counter these variable conditions and not compromise performance. Likewise, isolation of probiotic candidates from free-range poultry production systems may offer probiotic cultures with unique properties that could benefit a wide range of poultry production systems.

## References

[bib0001] Adhikari B., Kwon Y.M. (2017). Characterization of the culturable subpopulations of *Lactobacillus* in the chicken intestinal tract as a resource for probiotic development. Front. Microbiol..

[bib0002] Al-Qazzaz M.F.A., Ismail D., Akit H., Idris L.H. (2016). Effect of using insect larvae meal as a complete protein source on quality and productivity characteristics of laying hens. Rev. Bras. De Zootec..

[bib0003] Angelovičová M., Mellen M., Zdechovanová J. (2013). Applying the principles of welfare and quality of production in the organic farm of the laying hens. Potravinarstvo Slovak J. Food Sci.

[bib0004] Apata D. (2008). Growth performance, nutrient digestibility and immune response of broiler chicks fed diets supplemented with a culture of *Lactobacillus bulgaricus*. J. Sci. Food Agric..

[bib0006] Applegate T.J., Klose V., Steiner T., Ganner A., Schatzmayr G. (2010). Probiotics and phytogenics for poultry: Myth or reality?. J. Appl. Poult. Res..

[bib0007] Askelson T.E., Campasino A., Lee J.T., Duong T. (2014). Evaluation of phytate-degrading *Lactobacillus* culture administration to broiler chickens. Appl. Environ. Microbiol..

[bib0008] Aziz N.H., Khidhir Z., Hama Z.O., Mustafa N. (2020). Influence of probiotic (Miaclost) supplementation on carcass yield, chemical composition and meat quality of broiler chick. J. Appl. Poultry Prod..

[bib0009] Bager F., Aarestrup F.M., Madsen M., Wegener H.C. (1999). Glycopeptide resistance in *Enterococcus faecium* from broilers and pigs following discontinued use of avoparcin. Microb. Drug Resist..

[bib0010] Baines D., Sumarah M., Kuldau G., Juba J., Mazza A., Masson L. (2013). Aflatoxin, fumonisin and Shiga toxin-producing *Escherichia coli* infections in calves and the effectiveness of Celmanax®/Dairyman's ChoiceTM applications to eliminate morbidity and mortality losses. Toxins.

[bib0011] Bjerrum L., Engberg R., Leser T.D., Jensen B.B., Finster K., Pedersen K. (2006). Microbial community composition of the ileum and cecum of broiler chickens as revealed by molecular and culture-based techniques. Poult. Sci..

[bib0012] Bortoluzzi C., Serpa Vieira B., de Paula Dorigam J.C., Menconi A., Sokale A., Doranalli K., Applegate T.J. (2019). *Bacillus subtilis* DSM 32315 supplementation attenuates the effects of *Clostridium perfringens* challenge on the growth performance and intestinal microbiota of broiler chickens. Microorganisms.

[bib0013] Brisbin J.T., Gong J., Orouji S., Esufali J., Mallick A.I., Parvizi P., Shewen P.E., Sharif S. (2011). Oral treatment of chickens with lactobacilli influences elicitation of immune responses. Clin. Vaccine Immunol..

[bib0014] Bruce W., Corpet D. (1996). The colonic protein fermentation and insulin resistance hypotheses for colon cancer etiology: experimental tests using precursor lesions. Euro. J. Canc. Prev..

[bib0015] Bujalance C., Jiménez-Valera M., Moreno E., Ruiz-López M.-D., Lasserrot A., Ruiz-Bravo A. (2014). Lack of correlation between in vitro antibiosis and in vivo protection against enteropathogenic bacteria by probiotic lactobacilli. Res. Microbiol..

[bib0016] Buntyn J., Schmidt T., Nisbet D., Callaway T., Lewin H., Roberts R. (2016). The role of direct-fed microbials in conventional livestock production. Annu. Rev. Anim. Biosci..

[bib0017] Callaway T.R., Edrington T.S., Harvey R.B., Anderson R.C., Nisbet D.J. (2012). Prebiotics in food animals, a potential to reduce foodborne pathogens and disease. Rom. Biotechnol. Lett..

[bib0018] Callaway, T. R., T. S. Edrington, T. L. Poole, and D. J. Nisbet. 2011. Current status of practical applications: probiotics in dairy cattle. Pages 121–135 in Direct-Fed Microbials and Prebiotics For Animals: Science and Mechanisms of Action. S.C. Ricke and T.R. Callaway, eds. Springer, New York, NY.

[bib0019] Callaway, T., R. Anderson, T. Edrington, K. Genovese, R. Harvey, T. Poole, D. Nisbet, and J. Sofos. 2013. Novel methods for pathogen control in livestock pre-harvest: an update. Pages 275–304 in Advances in Microbial Food Safety. J. Sofos, ed. Woodhead Publishing, Cambridge, UK.

[bib0020] Callaway, T., T. Edrington, J. Byrd, and D. Nisbet. 2017. Use of direct-fed microbials in layer hen production-performance response and Salmonella control, producing safe eggs: microbial ecology of Salmonella. Pages 301–322 in Producing Safe Eggs. S. C. Ricke and R. K. Gast, eds. Academic Press, Cambridge, MA.

[bib0021] Casewell M., Friis C., Marco E., McMullin P., Phillips I. (2003). The European ban on growth-promoting antibiotics and emerging consequences for human and animal health. J. Antimicrob. Chemother..

[bib0022] Castellanos L.R., Donado-Godoy P., León M., Clavijo V., Arevalo A., Bernal J.F., Timmerman A.J., Mevius D.J., Wagenaar J.A., Hordijk J. (2017). High heterogeneity of *Escherichia coli* sequence types harbouring ESBL/AmpC genes on IncI1 plasmids in the Colombian poultry chain. PLoS One.

[bib0023] Castellini C., Mugnai C., Dal Bosco A. (2002). Effect of organic production system on broiler carcass and meat quality. Meat Sci.

[bib0024] Chang M.H., Chen T.C. (2000). Reduction of *Campylobacter jejuni* in a simulated chicken digestive tract by *Lactobacilli* cultures. J. Food Prot..

[bib0025] Cox C.M., Dalloul R.A. (2015). Immunomodulatory role of probiotics in poultry and potential *in ovo* application. Benef. Microbes.

[bib0026] Cui X., Marshall B., Shi N., Chen S.-Y., Rekaya R., Liu H.-X. (2017). RNA-Seq analysis on chicken taste sensory organs: An ideal system to study organogenesis. Sci. Rep..

[bib0027] De Preter V., Hamer H.M., Windey K., Verbeke K. (2011). The impact of pre- and/or probiotics on human colonic metabolism: does it affect human health?. Mol. Nutr. Food Res..

[bib0028] Ding J., Dai R., Yang L., He C., Xu K., Liu S., Zhao W., Xiao L., Luo L., Zhang Y., Meng H. (2017). Inheritance and establishment of gut microbiota in chickens. Front. Microbiol..

[bib0029] Dittoe D.K., McDaniel C.D., Tabler T., Kiess A.S. (2018). Windrowing poultry litter after a broiler house has been sprinkled with water. J. Appl. Poult. Res..

[bib0030] Dittoe, D.K., S.C. Ricke, and A.S. Kiess. 2020. Chapter 1. Commercial poultry production and gut function – Historical perspective. Pages. 3–30 in Improving Gut Function in Poultry. S.C. Ricke, ed. Burleigh Dodd Publishing, Cambridge, UK.

[bib0031] Dong X.F., Gao W.W., Tong J.M., Jia H.Q., Sa R.N., Zhang Q. (2007). Effect of polysavone (alfalfa extract) on abdominal fat deposition and immunity in broiler chickens. Poult. Sci..

[bib0032] Eeckhaut V., Wang J., Van Parys A., Haesebrouck F., Joossens M., Falony G., Raes J., Ducatelle R., Van Immerseel F. (2016). The probiotic *Butyricicoccus pullicaecorum* reduces feed conversion and protects from potentially harmful intestinal microorganisms and necrotic enteritis in broilers. Front. Microbiol..

[bib0034] El-Kholy A.M., El-Shinawy S.H., Meshref A.M.S., Korany A.M. (2014). Microbiological quality of Domiati cheese and the influence of probiotics on the behavior of *Staphylococcus aureus* and *Escherichia coli* O157:H7 in Domiati cheese. J. Food Safety.

[bib0035] Feng J., Wang L., Zhou L., Yang X., Zhao X. (2016). Using in vitro immunomodulatory properties of lactic acid bacteria for selection of probiotics against *Salmonella* infection in broiler chicks. PLoS One.

[bib0036] Feye K.M., Baxter M.F.A., Tellez G., Kogut M.H., Ricke S.C. (2020). Influential factors on the composition of the conventionally raised broiler gastrointestinal microbiomes. Poult. Sci..

[bib0037] Forte C., Acuti G., Manuali E., Proietti P., Pavone S., Trabalza M., Moscati L., Onofri A., Lorenzetti C., Franciosini M. (2016). Effects of two different probiotics on microflora, morphology, and morphometry of gut in organic laying hens. Poult. Sci..

[bib0038] Francis G., Kerem Z., Makkar H.P.S., Becker K. (2002). The biological action of saponins in animal systems: a review. Brit. J. Nutr..

[bib0039] Fricke W.F., McDermott P.F., Mammel M.K., Zhao S., Johnson T.J., Rasko D.A., Fedorka-Cray P.J., Pedroso A., Whichard J.M., LeClerc J.E., White D.G., Cebula T.A., Ravel J. (2009). Antimicrobial resistance-conferring plasmids with similarity to virulence plasmids from avian pathogenic *Escherichia coli* strains in *Salmonella enterica*/serovar Kentucky isolates from poultry. Appl. Environ. Microbiol..

[bib0040] Fuller R. (1989). Probiotics in man and animals. J. Appl. Bacteriol..

[bib0041] Furtula V., Farrell E.G., Diarrassouba F., Rempel H., Pritchard J., Diarra M.S. (2010). Veterinary pharmaceuticals and antibiotic resistance of *Escherichia coli* isolates in poultry litter from commercial farms and controlled feeding trials. Poult. Sci..

[bib0042] Gadde U., Kim W.H., Oh S.T., Lillehoj H.S. (2017). Alternatives to antibiotics for maximizing growth performance and feed efficiency in poultry: a review. Anim. Health Res. Rev..

[bib0043] Gaggìa F., Mattarelli P., Biavati B. (2010). Probiotics and prebiotics in animal feeding for safe food production. Int. J. Food Microbiol..

[bib0044] Ghareeb K., Awad W.A., Mohnl M., Porta R., Biarnés M., Böhm J., Schatzmayr G. (2012). Evaluating the efficacy of an avian-specific probiotic to reduce the colonization of *Campylobacter jejuni* in broiler chickens. Poult. Sci..

[bib0045] Ghareeb K., Awad W.A., Nitsch S., Abdel-Raheem S., Böhm J. (2008). Effects of transportation on stress and fear responses of growing broilers supplemented with prebiotic or probiotic. Int. J. Poult. Sci..

[bib0046] Gorbach S.L. (2000). Probiotics and gastrointestinal health. Am. J. Gastroenterol. 95(1 Suppl):S2-4.

[bib0047] Grigoroff, S. 1905. Etude sur le lait fermenté comestible: le “Kissélo-mléko” de Bulgarie. Revue médicale de la Suisse romande: organe officiel de la Société Médicale de la Suisse Romande, Lausanne, Switzerland.

[bib0048] Grilli E., Bari R., Piva A., Edrington T., Pitta D., Pinchak W., Nisbet D., Callaway T. (2015). Organic acid blend with pure botanical product treatment reduces *Escherichia coli* and *Salmonella* populations in pure culture and in in vitro mixed ruminal microorganism fermentations. Foodborne Pathog. Dis..

[bib0049] Hammershøj M., Johansen N.F. (2016). Review: the effect of grass and herbs in organic egg production on egg fatty acid composition, egg yolk colour and sensory properties. Livestock Sci.

[bib0050] Hao H., Cheng G., Iqbal Z., Ai X., Hussain H.I., Huang L., Dai M., Wang Y., Liu Z., Yuan Z. (2014). Benefits and risks of antimicrobial use in food-producing animals. Front. Microbiol..

[bib0051] He T., Long S., Mahfuz S., Wu D., Wang X., Wei X., Piao X. (2019). Effects of probiotics as antibiotics substitutes on growth performance, serum biochemical parameters, intestinal morphology, and barrier function of broilers. Animals.

[bib0052] Higgins J.P., Higgins S.E., Wolfenden A.D., Henderson S.N., Torres-Rodriguez A., Vicente J.L., Hargis B.M., Tellez G. (2010). Effect of lactic acid bacteria probiotic culture treatment timing on *Salmonella* Enteritidis in neonatal broilers. Poult. Sci..

[bib0053] Hoelzer K., Wong N., Thomas J., Talkington K., Jungman E., Coukell A. (2017). Antimicrobial drug use in food-producing animals and associated human health risks: what, and how strong, is the evidence?. BMC Vet. Res..

[bib0054] Holmberg S.D., Wells J.G., Cohen M.L. (1984). Animal-to-man transmission of antimicrobial-resistant *Salmonella*: investigations of U.S. outbreaks, 1971-1983. Science.

[bib0055] Hossain M.E., Kim G.M., Lee S.K., Yang C.J. (2012). Growth performance, meat yield, oxidative stability, and fatty acid composition of meat from broilers fed diets supplemented with a medicinal plant and probiotics. Asian-Austral. J. Anim. Sci..

[bib0056] Hussain Z., Khan M.Z., Khan A., Javed I., Saleemi M.K., Mahmood S., Asi M.R. (2010). Residues of aflatoxin B1 in broiler meat: Effect of age and dietary aflatoxin B1 levels. Food Chem. Toxicol..

[bib0057] Intanoo M., Kongkeitkajorn M.B., Pattarajinda V., Bernard J.K., Callaway T.R., Suriyasathaporn W., Phasuk Y. (2018). Isolation and screening of aflatoxin-detoxifying yeast and bacteria from ruminal fluids to reduce aflatoxin B1 contamination in dairy cattle feed. J. Appl. Microbiol..

[bib0058] Jin L.Z., Ho Y.W., Abdullah N., Jalaludin S. (1997). Probiotics in poultry: modes of action. World's Poult. Sci. J..

[bib0059] Jin L., Ho Y., Abdullah N., Jalaludin S. (1998). Growth performance, intestinal microbial populations, and serum cholesterol of broilers fed diets containing *Lactobacillus* cultures. Poult. Sci..

[bib0060] Joerger R.D. (2003). Alternatives to antibiotics: Bacteriocins, antimicrobial peptides and bacteriophages. Poult. Sci..

[bib0061] Karsten H.D., Patterson P.H., Stout R., Crews G. (2010). Vitamins A, E and fatty acid composition of the eggs of caged hens and pastured hens. Renew. Agr. Food Syst..

[bib0062] Katoch S., Sharma K.A., Chahota R., Sharma K.S., Radmila M., Šefer D. (2013). Performance of broiler chicken fed varied nutrient density diets supplemented with direct fed microbial. Acta Vet.

[bib0063] KeepAntibioticsWorking.com (2003). Antibiotic resistance - an emerging public health crisis. http://www.keepantibioticsworking.com/library/uploadedfiles/Antibiotic_Resistance_-_An_Emerging_Public_Hea.pdf.

[bib0064] Khan R., Naz S. (2013). The applications of probiotics in poultry production. World's Poult. Sci. J..

[bib0065] Kim E.Y., Kim Y.H., Rhee M.H., Song J.C., Lee K.W., Kim K.S., Lee S.P., Lee I.S., Park S.C. (2007). Selection of *Lactobacillus* sp. PSC101 that produces active dietary enzymes such as amylase, lipase, phytase and protease in pigs. J. Gen. Appl. Microbiol..

[bib0066] Kimura A.C., Reddy V., Marcus R., Cieslak P.R., Mohle-Boetani J.C., Kassenborg H.D., Segler S.D., Hardnett F.P., Barrett T., Swerdlow D.L. (2004). Chicken consumption is a newly identified risk factor for sporadic *Salmonella enterica* serotype enteritidis infections in the United States: A case-control study in FoodNet sites. Clin. Infect. Dis..

[bib0067] Kizerwetter-Swida M., Binek M. (2009). Protective effect of potentially probiotic *Lactobacillus* strain on infection with pathogenic bacteria in chickens. Pol. J. Vet. Sci.

[bib0068] Knap I., Kehlet A.B., Bennedsen M., Mathis G.F., Hofacre C.L., Lumpkins B.S., Jensen M.M., Raun M., Lay A. (2011). *Bacillus subtilis* (DSM17299) significantly reduces *Salmonella* in broilers. Poult. Sci..

[bib0069] Kogut M.H., Klasing K. (2009). An immunologist's perspective on nutrition, immunity, and infectious diseases: Introduction and overview. J.Appl. Poult. Res..

[bib0070] Kogut, M. H., and C. L. Swaggerty. 2011. Effects of prebiotics and probiotics on the host immune response, Pages 61–72 in Direct-Fed Microbials and Prebiotics For Animals: Science and Mechanisms of Action. S.C. Ricke and T.R. Callaway, eds. Springer, New York, NY.

[bib0071] Krehbiel C.R., Rust S.R., Zhang G., Gilliland S.E. (2003). Bacterial direct-fed microbials in ruminant diets: Performance response and mode of action. J. Anim. Sci..

[bib0072] La Ragione R.M., Casula G., Cutting S.M., Woodward M.J. (2001). *Bacillus subtilis* spores competitively exclude *Escherichia coli* O78:K80 in poultry. Vet. Microbiol..

[bib0073] La Ragione R.M., Narbad A., Gasson M.J., Woodward M.J. (2004). *In vivo* characterization of *Lactobacillus johnsonii* FI9785 for use as a defined competitive exclusion agent against bacterial pathogens in poultry. Let. Appl. Microbiol..

[bib0074] Lanzas C., Warnick L.D., James K.L., Wright E.M., Wiedmann M., GrÃhn Y.T. (2010). Transmission dynamics of a multidrug-resistant *Salmonella* Typhimurium outbreak in a dairy farm. Foodborne Path. Dis..

[bib0075] Lhermie G., Grohn Y.T., Raboisson D. (2016). Addressing antimicrobial resistance: an overview of priority actions to prevent suboptimal antimicrobial use in food-animal production. Front. Microbiol..

[bib0076] Li Z., Wang W., Liu D., Guo Y. (2018). Effects of *Lactobacillus acidophilus* on the growth performance and intestinal health of broilers challenged with *Clostridium perfringens*. J. Anim. Sci. Biotechnol..

[bib0077] Lilly D.M., Stillwell R.H. (1965). Probiotics: growth-promoting factors produced by microorganisms. Science.

[bib0078] Line J.E., Bailey J.S., Cox N.A., Stern N.J., Tompkins T. (1998). Effect of yeast-supplemented feed on *Salmonella* and *Campylobacter* populations in broilers. Poult. Sci..

[bib0079] Locatelli A., Hiett K.L., Caudill A.J., Rothrock M.J. (2017). Do fecal and litter microbiomes vary within the major areas of a commercial poultry house, and does this effect sampling strategies for whole house microbiomic studies?. J. Appl. Poul. Res..

[bib0080] Lokapirnasari W.P., Pribadi T.B., Al Arif A., Soeharsono S., Hidanah S., Harijani N., Najwan R., Huda K., Wardhani H.C.P., Rahman N.F.N. (2019). Potency of probiotics *Bifidobacterium* spp. and *Lactobacillus casei* to improve growth performance and business analysis in organic laying hens. Vet. World..

[bib0081] Lourenco J.M., Nunn S.C., Lee E., Dove C.R., Callaway T.R., Azain M.J. (2020). Effect of supplemental protease on growth performance and excreta microbiome of broiler chicks. Microorganisms.

[bib0082] Lourenco J.M., Rothrock M.J., Fluharty F.L., Callaway T.R. (2019). The successional changes in the gut microbiome of pasture-raised chickens fed soy-containing and soy-free diets. Front. Sustain. Food Syst..

[bib0083] Lourenco, J. M., D. S. Seidel, and T. R. Callaway. 2019b. Antibiotics and gut function: historical and current perspectives. Pages 172–189 in S.C. Ricke, editor, Improving Gut Health in Poultry. Francis Dodds Science Publishing, Cambridge, UK.

[bib0084a] Lu C.L., Baker. R.C. (1986). Characteristics of egg yolk phosvitin as an antioxidant for inhibiting metal-catalyzed phospholipid oxidations. Poult. Sci..

[bib0084] Lutful Kabir S.M. (2009). The role of probiotics in the poultry industry. Int. J. Molec. Sci..

[bib0085] Ma Q.G., Gao X., Zhou T., Zhao L.H., Fan Y., Li X.Y., Lei Y.P., Ji C., Zhang J.Y. (2012). Protective effect of Bacillus subtilis ANSB060 on egg quality, biochemical and histopathological changes in layers exposed to aflatoxin B1. Poult. Sci..

[bib0086] Maciorowski K., Herrera P., Jones F., Pillai S., Ricke S. (2007). Effects on poultry and livestock of feed contamination with bacteria and fungi. Anim. Feed Sci. Technol..

[bib0087] Manafi M., Khalaji S., Hedayati M. (2016). Assessment of a probiotic containing *Bacillus subtilis* on the performance and gut health of laying Japanese quails (Coturnix coturnix Japonica). Rev. Bras. Cienc..

[bib0088] Marshall B.M., Levy S.B. (2011). Food animals and antimicrobials: impacts on human health. Clin. Microbiol. Rev..

[bib0089] Mátéová S., Šály J., Tučková M., Koščová J., Nemcová R., Gaálová M., Baranová D. (2008). Effect of probiotics, prebiotics and herb oil on performance and metabolic parameters of broiler chickens. Med. Weter..

[bib0090] McAllister T.A., Beauchemin K.A., Alazzeh A.Y., Baah J., Teather R.M., Stanford K. (2011). Review: the use of direct fed microbials to mitigate pathogens and enhance production in cattle. Can. J. Anim. Sci..

[bib0091] Mench J., Sumner D., Rosen-Molina J. (2011). Sustainability of egg production in the United States—the policy and market context. Poult. Sci..

[bib0092] Metchnikoff, E. 1907. Lactic acid as inhibiting intestinal putrefaction. Pages 161–183 in The Prolongation of Life: Optimistic Studies. Heinemann, London, UK.

[bib0093] Miniello V., Diaferio L., Lassandro C., Verduci E. (2017). The importance of being eubiotic. J. Prob. Health.

[bib0094] Mortada M., Cosby D.E., Shanmugasundaram R., Selvaraj R.K. (2020). In vivo and in vitro assessment of commercial probiotic and organic acid feed additives in broilers challenged with *Campylobacter coli*. J. Appl. Poult. Res..

[bib0095] Motawe H.F.A., Abdel Salam A.F., El Meleigy K.M. (2014). Reducing the toxicity of aflatoxin in broiler chickens’ diet by using probiotic and yeast. Int. J. Poult. Sci..

[bib0096] Mountzouris K., Tsirtsikos P., Kalamara E., Nitsch S., Schatzmayr G., Fegeros K. (2007). Evaluation of the efficacy of a probiotic containing *Lactobacillus, Bifidobacterium, Enterococcus*, and *Pediococcus* strains in promoting broiler performance and modulating cecal microflora composition and metabolic activities. Poult. Sci..

[bib0097] Murate L.S., Paião F.G., de Almeida A.M., Berchieri A., Shimokomaki M. (2015). Efficacy of prebiotics, probiotics, and synbiotics on laying hens and broilers challenged with *Salmonella enteritidis*. Poult. Sci..

[bib0098] Nahashon S.N., Nakaue H.S., Mirosh L.W. (1994). Phytase activity, phosphorus and calcium retention, and performance of single comb White Leghorn layers fed diets containing two levels of available phosphorus and supplemented with direct-fed microbials. Poult. Sci..

[bib0099] Nahashon S.N., Nakaue H.S., Snyder S.P., Mirosh L.W. (1994). Performance of single comb White Leghorn layers fed corn-soybean meal and barley-corn-soybean meal diets supplemented with a direct-fed microbial. Poult. Sci..

[bib0100] Nava G.M., Bielke L.R., Callaway T.R., Castaneda M.P. (2005). Probiotic alternatives to reduce gastrointestinal infections: the poultry experience. Anim. Health Res. Rev..

[bib0101] Neveling D.P., Ahire J.J., Laubscher W., Rautenbach M., Dicks L.M. (2020). Genetic and phenotypic characteristics of a multi-strain probiotic for broilers. Curr. Microbiol..

[bib0102] Nunes R.V., Scherer C., Pozza P.C., Eyng C., Bruno L.D.G., Vieites F.M. (2012). Use of probiotics to replace antibiotics for broilers. Rev. Bras. De Zootec..

[bib0103] Nurmi E., Nuotio L., Schncitz C. (1992). The competitive exclusion concept: development and future. Int. J. Food Microbiol..

[bib0104] Olnood C.G., Beski S.S.M., Choct M., Iji P.A. (2015). Use of *Lactobacillus johnsonii* in broilers challenged with *Salmonella* Sofia. Anim. Nutr..

[bib0105] Otim M.O., Mukiibi-Muka G., Christensen H., Bisgaard M. (2005). Aflatoxicosis, infectious bursal disease and immune response to Newcastle disease vaccination in rural chickens. Avian Pathol.

[bib0106] Palamidi I., Fegeros K., Mohnl M., Abdelrahman W.H., Schatzmayr G., Theodoropoulos G., Mountzouris K.C. (2016). Probiotic form effects on growth performance, digestive function, and immune related biomarkers in broilers. Poult. Sci..

[bib0107] Pascual M., Hugas M., Badiola J.I., Monfort J.M., Garriga M. (1999). Lactobacillus salivarius CTC2197 prevents *Salmonella* Enteritidis colonization in chickens. Appl. Environ. Microbiol..

[bib0108] Patterson J.A., Burkholder K.M. (2003). Application of prebiotics and probiotics in poultry production. Poult. Sci..

[bib0109] Pelícia K., Mendes A.A., Saldanha E., Pizzolante C.C., Takahashi S.E., Garcia R.G. (2004). Probiotic and prebiotic utilization in diets for free-range broiler chickens. Rev. Bras. Ciênc. Avíc..

[bib0110] Penha Filho R.A.C., Díaz S.J.A., Fernando F.S., Chang Y.F., Andreatti Filho R.L., Berchieri Junior A. (2015). Immunomodulatory activity and control of *Salmonella* Enteritidis colonization in the intestinal tract of chickens by *Lactobacillus* based probiotic. Vet. Immunol. Immunopathol..

[bib0111] Perney K.M., Cantor A.H., Straw M.L., Herkelman K.L. (1993). The effect of dietary phytase on growth performance and phosphorus utilization of broiler chicks. Poult. Sci..

[bib0112] Ponte P.I.P., Rosado C.M.C., Crespo J.P., Crespo D.G., Mourão J.L., Chaveiro-Soares M.A., Brás J.L.A., Mendes I., Gama L.T., Prates J.A.M., Ferreira L.M.A., Fontes C.M.G.A. (2008). Pasture intake improves the performance and meat sensory attributes of free-range broilers. Poult Sci.

[bib0113] Poole T.L., Callaway T.R., Norman K.N., Scott H.M., Loneragan G.H., Ison S.A., Beier R.C., Harhay D.M., Norby B., Nisbet D.J. (2017). Transferability of antimicrobial resistance from multidrug-resistant *Escherichia coli* isolated from cattle in the USA to *E. coli* and *Salmonella* Newport recipients. J. Glob. Antimicrob. Resist..

[bib0114] Ramlucken U., Ramchuran S.O., Moonsamy G., Lalloo R., Thantsha M.S., Jansen van Rensburg C. (2020). A novel *Bacillus* based multi-strain probiotic improves growth performance and intestinal properties of *Clostridium perfringens* challenged broilers. Poult. Sci..

[bib0115] Randall L.P., Ridley A.M., Cooles S.W., Sharma M., Sayers A.R., Pumbwe L., Newell D.G., Piddock L.J.V., Woodward M.J. (2003). Prevalence of multiple antibiotic resistance in 443 *Campylobacter* spp. isolated from humans and animals. J. Antimicrob. Chemother..

[bib0116] Rao A.V., Gurfinkel D.M. (2000). The bioactivity of saponins: triterpenoid and steroidal glycosides. Drug Metabol. Person. Ther..

[bib0117] Ricke S.C. (2017). Insights and challenges of *Salmonella* infection of laying hens. Curr. Opin. Food Sci..

[bib0118] Ricke S.C., Lee S.I., Kim S.A., Park S.H., Shi Z. (2020). Prebiotics and the poultry gastrointestinal tract microbiome. Poult. Sci..

[bib0119] Ricke S.C., Rothrock M.J. (2020). Gastrointestinal microbiomes of broilers and layer hens in alternative production systems. Poult. Sci..

[bib0120] Rinttilä T., Apajalahti J. (2013). Intestinal microbiota and metabolites—Implications for broiler chicken health and performance. J. Appl. Poult. Res..

[bib0121] Rodjan P., Soisuwan K., Thongprajukaew K., Theapparat Y., Khongthong S., Jeenkeawpieam J., Salaeharae T. (2018). Effect of organic acids or probiotics alone or in combination on growth performance, nutrient digestibility, enzyme activities, intestinal morphology and gut microflora in broiler chickens. J. Anim. Physiol. Anim. Nutr..

[bib0122] Rothrock M.J., Gibson K.E., Micciche A.C., Ricke S.C. (2019). Pastured poultry production in the united states: strategies to balance system sustainability and environmental impact. Front. Sustain. Food Syst..

[bib0123] Rotz C.A. (2004). Management to reduce nitrogen losses in animal production. J. Anim. Sci..

[bib0124] Rowghani E., Arab M., Akbarian A. (2007). Effects of a probiotic and other feed additives on performance and immune response of broiler chicks. Int. J. Poult. Sci..

[bib0125] Ruhnke I., Normant C., Campbell D.L.M., Iqbal Z., Lee C., Hinch G.N., Roberts J. (2018). Impact of on-range choice feeding with black soldier fly larvae (*Hermetia illucens*) on flock performance, egg quality, and range use of free-range laying hens. Anim. Nutr..

[bib0126] Saint-Cyr M.J., Haddad N., Taminiau B., Poezevara T., Quesne S., Amelot M., Daube G., Chemaly M., Dousset X., Guyard-Nicodème M. (2017). Use of the potential probiotic strain *Lactobacillus salivarius* SMXD51 to control *Campylobacter jejuni* in broilers. Int. J. Food Microbiol..

[bib0127] Schneitz C. (2005). Competitive exclusion in poultry–30 years of research. Food Cont.

[bib0128] Seal B.S., Lillehoj H.S., Donovan D.M., Gay C.G. (2013). Alternatives to antibiotics: a symposium on the challenges and solutions for animal production. Anim. Health Res. Rev..

[bib0129] Shi Z., Rothrock M.J., Ricke S.C. (2019). Applications of microbiome analyses in alternative poultry broiler production systems. Front. Vet. Sci..

[bib0130] Shini S., Shini A., Blackall P. (2013). The potential for probiotics to prevent reproductive tract lesions in free-range laying hens. Anim. Prod. Sci..

[bib0131] Siemon C.E., Bahnson P.B., Gebreyes W.A. (2007). Comparative investigation of prevalence and antimicrobial resistance of *Salmonella* between pasture and conventionally reared poultry. Avian Dis.

[bib0132] Smialek M., Burchardt S., Koncicki A. (2018). The influence of probiotic supplementation in broiler chickens on population and carcass contamination with *Campylobacter* spp. - field study. Res. Vet. Sci..

[bib0133] Sokale A.O., Menconi A., Mathis G.F., Lumpkins B., Sims M.D., Whelan R.A., Doranalli K. (2019). Effect of *Bacillus subtilis* DSM 32315 on the intestinal structural integrity and growth performance of broiler chickens under necrotic enteritis challenge. Poult. Sci..

[bib0134] Sossidou E., Bosco A., Castellini C., Grashorn M. (2015). Effects of pasture management on poultry welfare and meat quality in organic poultry production systems. World's Poult. Sci. J..

[bib0135] Soupir M.L., Mostaghimi S., Yagow E.R., Hagedorn C., Vaughan D.H. (2006). Transport of fecal bacteria from poultry litter and cattle manures applied to pastureland. Water Air Soil Poll.

[bib0136] Suvarna V., Boby V. (2005). Probiotics in human health: a current assessment. Curr. Sci..

[bib0137] Swaggerty C.L., Callaway T.R., Kogut M.H., Piva A., Grilli E. (2019). Modulation of the immune response to improve health and reduce foodborne pathogens in poultry. Microorganisms.

[bib0138] Sweeney M.T., Lubbers B.V., Schwarz S., Watts J.L. (2018). Applying definitions for multidrug resistance, extensive drug resistance and pandrug resistance to clinically significant livestock and companion animal bacterial pathogens. J. Antimicrob. Chemother..

[bib0139] Taheri H.R., Moravej H., Tabandeh F., Zaghari M., Shivazad M. (2010). Efficacy of combined or single use of *Lactobacillus crispatus* lt116 and *L. johnsonii* lt171 on broiler performance. Br. Poult. Sci..

[bib0140] Takahashi S., Mendes A.A., Saldanha E., Pizzolante C., Pelícia K., Quinteiro R., Komiyama C., Garcia R., Almeida Paz I. (2005). Efficiency of prebiotics and probiotics on the performance, yield, meat quality and presence of *Salmonella* spp in carcasses of free-range broiler chickens. Rev. Bras. Cienc..

[bib0141] Tannock G.W. (1997). Probiotic properties of lactic-acid bacteria: plenty of scope for fundamental R & D. Trends Biotechnol.

[bib0142] Tarus J., Rachuonyo H., Omega J., Ochuodho J. (2019). Assessment of aflatoxin levels in indigenous chicken tissues and eggs in Western Kenya. African J. Education, Sci. and Technol..

[bib0143] Trembecká L., Haščík P., Čuboň J., Bobko M., Pavelková A. (2016). Fatty acids profile of breast and thigh muscles of broiler chickens fed diets with propolis and probiotics. J. Cent. Euro. Agric..

[bib0144] Ungemach F.R., Muller-Bahrdt D., Abraham G. (2006). Guidelines for prudent use of antimicrobials and their implications on antibiotic usage in veterinary medicine. Int. J. Med. Microbiol..

[bib0145] Upadhaya S.D., Hossiendoust A., Kim I.H. (2016). Probiotics in Salmonella-challenged Hy-Line brown layers. Poult. Sci..

[bib0146] USDA-FSIS (2007). Progress Report on Salmonella Testing of Raw Meat and Poultry Products, 1998–2006. http://www.fsis.usda.gov/Science/Progress_Report_Salmonella_Testing_Tables/index.asp.

[bib0147a] US Egg and Poultry. 2020. US Hen Facts and Statistics for 2020. Accessed April 2021. https://unitedegg.com/facts-stats/.

[bib0147] Van Boeckel T.P., Brower C., Gilbert M., Grenfell B.T., Levin S.A., Robinson T.P., Teillant A., Laxminarayan R. (2015). Global trends in antimicrobial use in food animals. Proc. Nat. Acad. Sci. (USA).

[bib0148] Vandana R., Brijesh Y., Lakhani G.P. (2013). Application of probiotic and prebiotic in animals production: a review. Environ. Ecol..

[bib0149] Vase-Khavari K., Mortezavi S.H., Rasouli B., Khusro A., Salem A.Z.M., Seidavi A. (2019). The effect of three tropical medicinal plants and superzist probiotic on growth performance, carcass characteristics, blood constitutes, immune response, and gut microflora of broiler. Trop. Anim. Health Prod..

[bib0150] Vasiljevic T., Shah N.P. (2008). Probiotics—from Metchnikoff to bioactives. Int. Dairy J..

[bib0151] Wang H., Lee I.-S., Braun C., Enck P. (2016). Effect of probiotics on central nervous system functions in animals and humans: a systematic review. J Neurogastroenterol. Motil..

[bib0152] Wang L., Fang M., Hu Y., Yang Y., Yang M., Chen Y. (2014). Characterization of the most abundant *Lactobacillus* species in chicken gastrointestinal tract and potential use as probiotics for genetic engineering. Acta. Biochim. Biophys. Sin..

[bib0153] Williams-Nguyen J., Sallach J.B., Bartelt-Hunt S., Boxall A.B., Durso L.M., McLain J.E., Singer R.S., Snow D.D., Zilles J.L. (2016). Antibiotics and antibiotic resistance in agroecosystems: state of the science. J Environ Qual.

[bib0154] Willis W.L., Reid L. (2008). Investigating the effects of dietary probiotic feeding regimens on broiler chicken production and *Campylobacter jejuni* presence. Poult. Sci..

[bib0155] Wray C., Davies R.H. (2000). Competitive exclusion-an alternative to antibiotics. Vet. J..

[bib0156] Wu Y., Zhen W., Geng Y., Wang Z., Guo Y. (2019). Pretreatment with probiotic *Enterococcus faecium* NCIMB 11181 ameliorates necrotic enteritis-induced intestinal barrier injury in broiler chickens. Sci. Rep..

[bib0157] Yaşar S., Okutan İ., Tosun R. (2017). Testing novel eubiotic additives: its health and performance effects in commercially raised farm animals. Iğdır Univ. J. Inst. Sci. Tech..

[bib0158] Yazhini P., Visha P., Selvaraj P., Vasanthakumar P., Chandran V. (2018). Dietary encapsulated probiotic effect on broiler serum biochemical parameters. Vet. World.

[bib0159] Yeo J., Kim K.I. (1997). Effect of feeding diets containing an antibiotic, a probiotic, or yucca extract on growth and intestinal urease activity in broiler chicks. Poult. Sci..

[bib0160] Żakowska-Biemans S., Tekień A. (2017). Free range, organic? polish consumers preferences regarding information on farming system and nutritional enhancement of eggs: a discrete choice based experiment. Sustainability.

[bib0161] Zhou M., Zeng D., Ni X., Tu T., Yin Z., Pan K., Jing B. (2016). Effects of *Bacillus licheniformis* on the growth performance and expression of lipid metabolism-related genes in broiler chickens challenged with *Clostridium perfringens-*induced necrotic enteritis. Lipids Health Dis.

